# The Role of Infrared Thermography in the Diagnosis and Monitoring of Diabetic Foot Infection: A Systematic Review

**DOI:** 10.3390/idr18050098

**Published:** 2026-09-07

**Authors:** Carmen Cantarero-Lozano, Aroa Tardáguila-García, Esther García-Morales, Yolanda García-Álvarez, David Navarro-Pérez, José Luis Lázaro-Martínez

**Affiliations:** 1 Diabetic Foot Unit, Clínica Universitaria de Podología, Facultad de Enfermería, Fisioterapia y Podología, Instituto de Investigación Sanitaria del Hospital Clínico San Carlos (IdISSC), Universidad Complutense de Madrid, 28040 Madrid, Spain; carcanta@ucm.es (C.C.-L.); eagarcia@ucm.es (E.G.-M.); ygarci01@ucm.es (Y.G.-Á.); diabetes@ucm.es (J.L.L.-M.); 2 Podosauces Podiatry and Health, 03006 Alicante, Spain; davinava@ucm.es

**Keywords:** thermography, infrared thermography, diabetic foot, diabetic foot infection, diabetic foot ulcer

## Abstract

Background: Diabetic foot infection (DFI) is one of the most frequent diabetes-related complications requiring hospitalisation and is a major contributor to lower-extremity amputation. Infrared thermography has emerged as a non-invasive diagnostic tool capable of detecting temperature changes associated with inflammatory and infectious processes. This systematic review aimed to evaluate the role of thermography in the diagnosis and monitoring of diabetic foot infection. Methods: The Preferred Reporting Items for Systematic Reviews and Meta-Analyses (PRISMA) statement was followed. Risk of bias and methodological quality were assessed using design-specific validated tools, including QUADAS-2 for diagnostic and detection studies and Joanna Briggs Institute critical appraisal checklists for cohort and case-series studies. Original studies evaluating the use of thermography in patients with diabetic foot infection were included. Two authors independently performed study selection, data extraction, and methodological assessment. Results: Six studies met the inclusion criteria, comprising a total of 552 participants with type 1 or type 2 diabetes mellitus. Four observational studies and two pilot studies were included. Increased local skin temperature was consistently associated with acute diabetic foot complications and infection. Several studies identified a temperature difference of approximately 2.2 °C between contralateral foot regions as a clinically relevant threshold for detecting diabetic foot complications. However, no significant relationship was found between plantar thermal asymmetry and the severity or progression of infected diabetic foot ulcers. All included studies presented a level of evidence of 4 and a grade of recommendation of C. Conclusions: Infrared thermography appears to be a promising adjunctive tool for the early detection of diabetic foot infection. However, current evidence does not support its routine use for monitoring infection severity, treatment response, or prognosis, and further prospective studies are required before these applications can be recommended.

## 1. Introduction

Diabetes mellitus is one of the most prevalent chronic diseases worldwide and is associated with multiple microvascular and macrovascular complications. Among these, diabetic foot disease represents one of the most serious and costly complications, significantly increasing morbidity, disability, healthcare expenditure, and mortality [1,2,3,4,5].

The International Working Group on the Diabetic Foot (IWGDF) defines diabetic foot disease as infection, ulceration, or destruction of foot tissues associated with peripheral neuropathy and/or peripheral arterial disease in a person with diabetes mellitus [1,2]. Diabetic foot ulceration (DFU) affects approximately 19–34% of individuals with diabetes during their lifetime and is associated with high recurrence rates, reaching 40% within one year and 65% within three years after healing [4,5].

Diabetic foot infection (DFI) remains one of the most common diabetes-related complications requiring hospitalisation and is a major precipitating factor for lower-extremity amputation [6,7]. Early diagnosis and treatment are essential to reduce tissue destruction, prevent amputations, and improve patient outcomes.

Several studies have demonstrated that temperature alterations are associated with diabetic foot complications [8,9]. Inflammatory and infectious processes increase local metabolic activity and blood flow, resulting in measurable changes in skin temperature. Consequently, thermal assessment has emerged as a potential adjunctive diagnostic tool.

Infrared thermography is a non-invasive imaging technique that measures infrared radiation emitted from the skin surface and converts these measurements into temperature maps [10,11,12]. The technique is painless, rapid, and suitable for repeated assessments. Previous research has suggested that thermal asymmetry between corresponding regions of the feet may represent an early marker of tissue stress, ulceration, inflammation, and infection [13].

The use of infrared thermography in diabetic foot care has increased considerably over recent years due to advances in thermal imaging technology and image-processing software [9]. The technique offers objective and reproducible measurements of skin temperature and has the potential to support early diagnosis, risk stratification, and remote monitoring of diabetic foot complications. Furthermore, its non-invasive nature and absence of radiation exposure make it an attractive option for repeated clinical assessments.

Despite increasing interest in thermographic assessment, the evidence regarding its specific role in the diagnosis and monitoring of DFI remains limited and heterogeneous. Although a recent systematic review and meta-analysis by García-de-la-Peña et al. (2024) [14] evaluated the use of infrared thermography across a broad spectrum of podiatric conditions, including diabetic foot ulcer prevention, peripheral arterial disease, musculoskeletal disorders and onychomycosis, no previous review has specifically synthesized the available evidence regarding its role in the diagnosis and monitoring of diabetic foot infection. Given the distinct pathophysiological mechanisms, diagnostic challenges and clinical implications of infection compared with other diabetic foot complications, a focused systematic review is warranted to critically appraise the current evidence and identify existing knowledge gaps. Consequently, the clinical applicability of thermography in the management of DFI remains uncertain.

Therefore, the aim of this systematic review was to evaluate the available evidence regarding the role of infrared thermography in the diagnosis and monitoring of diabetic foot infection, with particular emphasis on its diagnostic utility, clinical applicability, and potential value in predicting disease progression and treatment response.

## 2. Materials and Methods

### 2.1. Study Design

A systematic review was conducted to evaluate the role of infrared thermography in the diagnosis and monitoring of diabetic foot infection (DFI). The methodology followed the recommendations of the Preferred Reporting Items for Systematic Reviews and Meta-Analyses (PRISMA) statement [15]. The PRISMA 2020 checklist, including the location of each item within the manuscript, is provided in Supplementary Table S1 [16].

The research question was developed according to the Population, Intervention, Comparison and Outcome (PICO) framework (Table 1). The objective of the review was to determine whether thermographic assessment could facilitate the diagnosis of diabetic foot infection and identify thermal patterns associated with diabetic foot complications.

### 2.2. Literature Search Strategy

A comprehensive literature search was conducted using the electronic databases PubMed (MEDLINE) and Scopus.

The search strategy combined controlled vocabulary (Mesh terms, where applicable) and free-text keywords related to diabetic foot and infrared thermography. The following search strategy was initially developed for PubMed and subsequently adapted to the syntax of the remaining databases: (“Diabetic Foot” [Mesh] OR diabetic foot OR diabetic foot ulcer OR foot ulcer) AND (“Thermography” [Mesh] OR thermograph* OR infrared thermograph* OR thermal imag*) AND (infect* OR osteomyelitis OR soft tissue infection*). Boolean operators (AND/OR) were used to maximise search sensitivity while maintaining relevance to the review question.

Because studies involving diabetic foot infection are frequently indexed or described using broader terms such as diabetic foot or diabetic foot ulcer, a deliberately sensitive search strategy was employed. Eligibility was subsequently determined through title/abstract and full-text screening according to the predefined PICO criteria.

No restrictions regarding study design were initially applied during the search process. In addition, manual searches of the reference lists of relevant articles were conducted to identify potentially eligible studies not retrieved through the electronic databases.

### 2.3. Eligibility Criteria

Studies were selected according to predefined inclusion and exclusion criteria.

Inclusion Criteria: Studies were considered eligible if they met the following criteria: original research articles; clinical trials, pilot studies; randomised clinical trials or observational studies; studies evaluating the use of thermography in patients with diabetic foot infection; studies investigating the diagnostic or monitoring value of thermographic assessment; and articles published in English, Spanish, French, German or Italian.

Exclusion Criteria: Studies were excluded if they met any of the following criteria: systematic reviews, narrative reviews or meta-analyses; studies not specifically related to diabetic foot disease; studies not evaluating thermography as a diagnostic or monitoring tool; studies providing insufficient information for data extraction; and editorials, conference abstracts, letters to the editor or expert opinion articles. The studies were also excluded if they provided insufficient data for extraction or reported outcomes that were not relevant to the objectives of the present review.

### 2.4. Study Selection

Two reviewers (C.C.L. and A.T.G.) independently screened the titles and abstracts of all retrieved records. Full-text articles were subsequently assessed for eligibility according to the predefined inclusion and exclusion criteria.

Any disagreement between reviewers was resolved through discussion and consensus.

**Figure 1:**
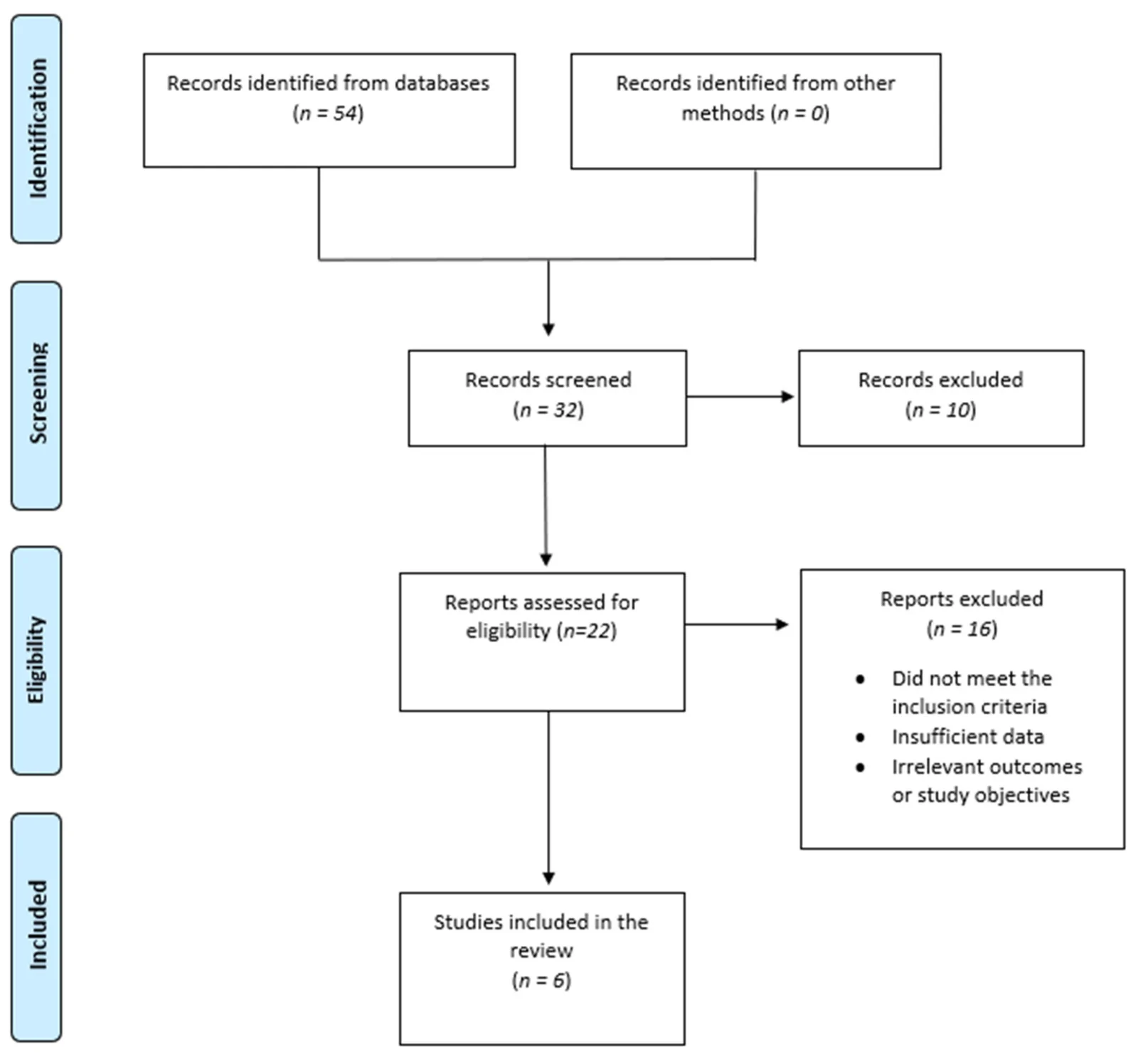
PRISMA 2020 flow diagram illustrating the identification, screening, eligibility assessment, and inclusion of studies in the systematic review.

The study selection process is presented in the PRISMA flow diagram (Figure 1).

### 2.5. Data Extraction

Data extraction was independently performed by the same two reviewers using a standardised data collection form developed specifically for this review.

The following variables were extracted from each study: first author; year of publication; number of participants; study design; diabetic foot classification system; type of thermographic assessment; evaluated parameters; study objectives; and main findings.

The characteristics of the included studies are summarised in Table 2.

### 2.6. Risk-of-Bias and Methodological Quality Assessment

Risk of bias and methodological quality were assessed independently by two reviewers using validated tools selected according to the design and primary objective of each included study. Studies evaluating the diagnostic or detection performance of infrared thermography were assessed using the Quality Assessment of Diagnostic Accuracy Studies-2 (QUADAS-2) tool [22] QUADAS-2 evaluates risk of bias across four domains: patient selection, index test, reference standard, and flow and timing. Concerns regarding applicability were also assessed for the patient selection, index test, and reference standard domains. Each domain was classified as having low, unclear, or high risk of bias or applicability concern.

The study by Armstrong et al. [19], which prospectively evaluated the association between dermal temperature measurements and subsequent clinical outcomes, was assessed using the Joanna Briggs Institute Critical Appraisal Checklist for Cohort Studies [23]. The prospective case series by Hutting et al. [21] was assessed using the Joanna Briggs Institute Critical Appraisal Checklist for Case Series.

**Figure 2:**
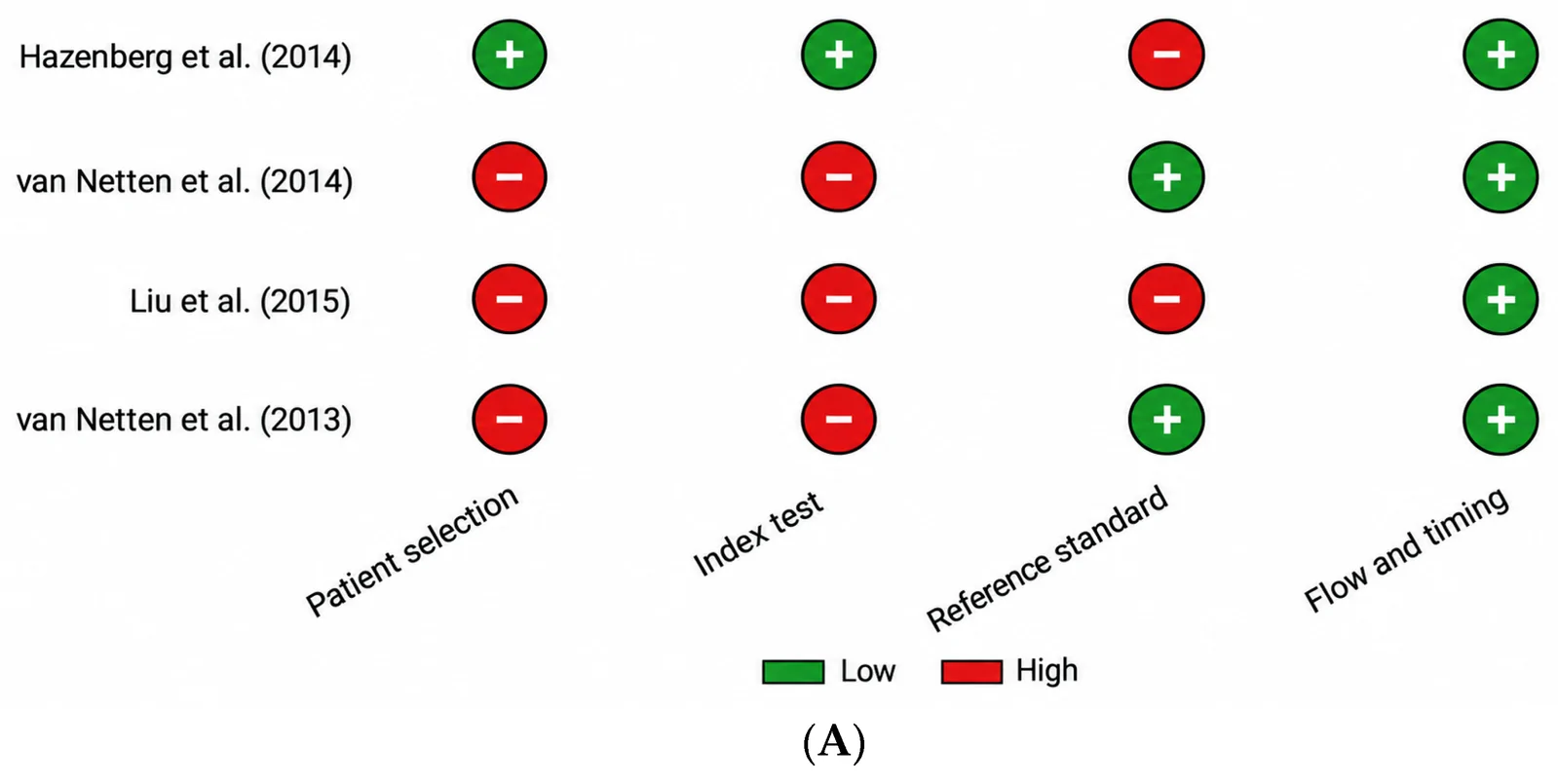

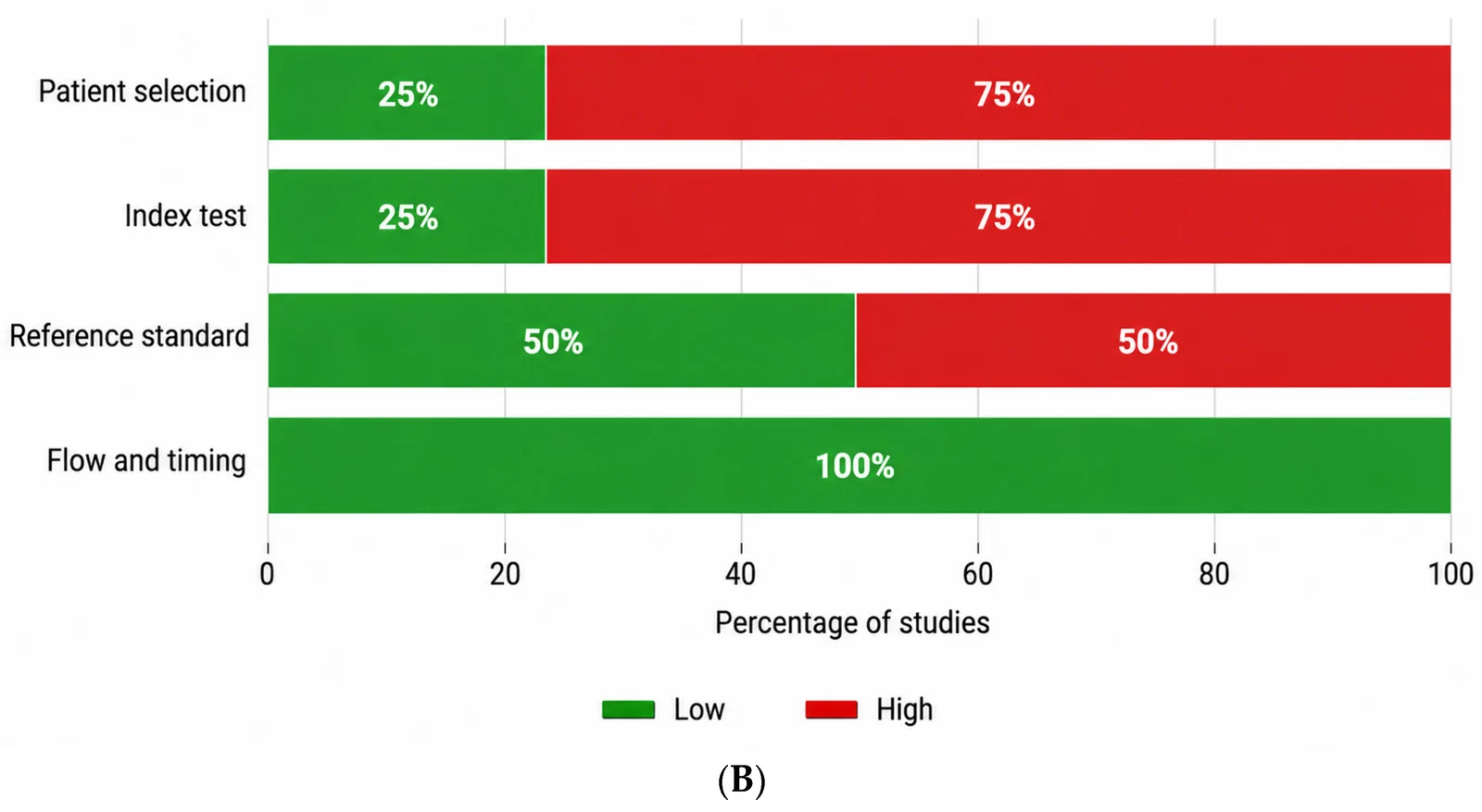
Risk-of-bias assessment of studies evaluating the diagnostic or detection performance of infrared thermography using the QUADAS-2 tool [22]. (**A**) QUADAS-2 risk-of-bias summary. Study-level risk-of-bias judgements across the patient selection, index test, reference standard, and flow and timing domains [11,17,18,20]. (**B**) QUADAS-2 risk-of-bias graph. Proportion of studies classified as having low, unclear, or high risk of bias in each QUADAS-2 domain. Green indicates low risk of bias, yellow indicates unclear risk of bias, and red indicates high risk of bias.

Domain-level judgements were considered individually in the interpretation of the evidence. No overall numerical quality score or arbitrary cut-off value was applied. Any disagreement between the two reviewers was resolved through discussion and consensus. The QUADAS-2 assessment is presented in Figure 2, whereas the assessment of the monitoring studies is summarised in Table 3 and reported in detail in Supplementary Tables S2 and S3.

### 2.7. Levels of Evidence and Grade of Recommendation

The level of evidence and grade of recommendation of the included studies were classified according to the Oxford Centre for Evidence-Based Medicine (OCEBM) Levels of Evidence framework [24].

Additionally, the Science Citation Index Expanded (SCIE) quartile ranking and journal impact factor of each study were recorded (Table 4).

### 2.8. Outcome Measures

The primary outcome of this review was the usefulness of thermography for identifying thermal alterations associated with diabetic foot infection.

Secondary outcomes included:

1. Detection of temperature differences associated with clinical signs of infection, including inflammation, erythema, ulceration and tissue damage.
2. Association between thermographic patterns and diabetic foot complications.
3. Utility of thermography for monitoring infection severity and treatment response.

Due to the heterogeneity of the included studies regarding design, thermographic methodology, and outcome measures, a quantitative meta-analysis was not performed.

### 2.9. Protocol Registration

The protocol for this systematic review was not registered. However, the review methodology was prespecified prior to the initiation of the review process and was con-ducted in accordance with the PRISMA guidelines [15].

## 3. Results

### 3.1. Study Selection

The electronic database search identified a total of 54 records. After removal of duplicate studies and screening of titles and abstracts, 22 articles were considered potentially eligible and underwent full-text assessment. Following detailed evaluation, 16 studies were excluded because they did not meet the predefined inclusion criteria. Consequently, six studies were included in the final qualitative synthesis.

Following the updated literature search performed in June 2026, additional records were screened. However, no further studies fulfilled the predefined eligibility criteria, and therefore no additional studies were included in the review.

The study selection process is summarised in the PRISMA flow diagram (Figure 1).

### 3.2. Characteristics of the Included Studies

The six included studies were published between 2006 and 2020 and involved a total of 552 participants with type 1 or type 2 diabetes mellitus. Four studies were observational studies and two were pilot studies.

All included studies presented a level of evidence of 4 and a grade of recommendation of C according to the Oxford Centre for Evidence-Based Medicine classification (Table 2).

The methodological quality of the included studies was evaluated using the QUADAS-2 tool. Overall, the included studies showed a low-to-moderate risk of bias. The main concerns were related to patient selection, owing to convenience sampling and small sample sizes, whereas the index test and reference standard domains generally showed a low risk of bias. Flow and timing were adequately reported in most studies. Figure 2A,B summarize the QUADAS-2 assessment.

The main characteristics of the included studies, including sample size, study design, thermographic methodology, evaluated parameters, and principal findings, are summarised in Table 5.

The included studies investigated different applications of infrared thermography in diabetic foot disease, including the detection of diabetic foot infection, identification of diabetic foot complications, assessment of thermal asymmetry, and monitoring of treatment response.

### 3.3. Diagnostic and Prognostic Value of Infrared Thermography

Hazenberg et al. [17] evaluated 38 patients using photographic foot imaging combined with infrared thermography and reported that thermographic assessment was a valid and reliable method for identifying signs of diabetic foot infection. The authors concluded that infrared thermography may improve diagnostic assessment, particularly in patients where clinical examination is limited by neuropathy or structural deformities.

Similarly, Van Netten et al. [18] assessed the diagnostic value of skin temperature measurements in patients with diabetic foot complications. The authors reported that a temperature difference of 2.2 °C between corresponding contralateral foot regions represented the optimal threshold for detecting diabetes-related foot complications. Furthermore, a mean temperature difference of 1.35 °C between feet was identified as a useful indicator of treatment urgency.

Liu et al. [11] evaluated 76 patients using infrared thermography and automated thermal asymmetry analysis. Using a threshold of 2.2 °C between corresponding areas of both feet, the authors successfully identified 35 of 37 diabetic foot ulcers, corresponding to a detection rate of approximately 95%. These findings support the potential role of thermal asymmetry as an objective marker of diabetic foot complications.

#### 3.3.1. Thermographic Patterns Associated with Diabetic Foot Complications

Van Netten et al. [20] conducted a pilot study involving 15 patients divided into three groups according to the severity of diabetic foot complications.

Patients without visible complications showed no significant temperature differences between feet. In contrast, patients presenting local complications such as hyperkeratosis or neuropathic ulceration demonstrated temperature increases greater than 2 °C in the affected region. Patients with diffuse complications, including infected ulcers, Charcot neuroarthropathy, and critical limb ischaemia, showed temperature differences exceeding 3 °C between feet.

These findings suggest that increasing thermal asymmetry may be associated with the severity and extent of diabetic foot pathology.

Similarly, van Netten et al. [18] evaluated the diagnostic performance of infrared thermography for the detection of diabetes-related foot complications by analysing mean temperature differences between corresponding regions of the feet. Although a cut-off value of 1.35 °C was identified as the optimal threshold, the authors concluded that the detection of diabetes-related foot complications based solely on local skin temperature assessment was limited by relatively low diagnostic accuracy. These findings suggest that thermal asymmetry should be interpreted as an adjunctive indicator rather than a standalone diagnostic criterion for diabetic foot pathology.

#### 3.3.2. Thermography for Monitoring Infection Severity and Treatment Response

The usefulness of thermography for monitoring diabetic foot infection remains less clear.

Hutting et al. [21] evaluated seven patients with infected plantar diabetic foot ulcers and investigated whether plantar thermal asymmetry could be used to monitor infection severity and treatment response. No significant relationship was observed between thermal asymmetry and either infection severity or ulcer progression. Consequently, the authors concluded that infrared thermography may have limited value as a monitoring tool, although it may still provide diagnostic or prognostic information.

Armstrong et al. [19] analysed data from 362 patients included in the SIDESTEP trial and investigated the relationship between skin temperature differences and systemic inflammatory markers, including leukocyte count, C-reactive protein, erythrocyte sedimentation rate, and Texas wound classification. No significant correlation was identified between baseline skin temperature differences and systemic inflammatory markers. However, thermometric assessment remained useful for monitoring local tissue changes during clinical follow-up.

### 3.4. Risk-of-Bias and Methodological Quality Assessment

Four studies evaluating the diagnostic or detection performance of infrared thermography were assessed using the QUADAS-2 tool (University of Bristol, Bristol, United Kingdom). The main methodological concerns were identified in the patient selection and index test domains. Three of the four studies were judged to have a high risk of selection bias because they used convenience samples, purposively selected populations, or small groups defined according to the presence and severity of diabetic foot complications.

Three studies were judged to have a high risk of bias in the index test domain because thermal thresholds or image-analysis algorithms were developed and evaluated using the same study population without independent external validation. Two studies presented a high risk of bias in the reference standard domain. In Hazenberg et al. [17], the clinical reference standard showed only moderate interobserver agreement, whereas in Liu et al. [11], manual image annotation was an appropriate technical reference for image segmentation but did not constitute an independent clinical reference standard for diabetic foot infection. Flow and timing were judged to have a low risk of bias in all four studies. The study-level and domain-level QUADAS-2 judgements are presented in Figure 2.

Applicability concerns were predominantly related to patient selection. All four studies included heterogeneous populations with diabetic foot complications rather than populations composed exclusively of patients with diabetic foot infection. Additional concerns were identified in Liu et al. [11] because the primary objective was the technical validation of image segmentation and registration rather than the clinical diagnosis of diabetic foot infection. The complete applicability assessment is presented in Figure 1 and the supporting domain-level judgements are provided in Supplementary Table S4.

The two studies primarily focused on monitoring infection severity or treatment response were evaluated using design-specific Joanna Briggs Institute critical appraisal tools. Armstrong et al. [19] showed methodological strengths related to its large multicentre sample, standardised thermometry protocol, investigator training, and high proportion of participants with follow-up measurements. Nevertheless, concerns arose from the secondary nature of the analysis, incomplete adjustment for potential confounding factors, absence of thermometric measurements at completion of the full treatment course, and the exploratory assessment of the 10 °F threshold.

Hutting et al. [21] clearly reported its eligibility criteria, thermographic acquisition protocol, clinical assessment procedures, and study outcomes. However, the use of a convenience sample, the absence of demonstrated consecutive or complete recruitment, and the inclusion of only seven participants substantially limited the precision and generalisability of its findings. The assessment of both monitoring studies is summarised in Table 3 and reported in detail in Supplementary Tables S2 and S3.

### 3.5. Overall Findings

Overall, the available evidence suggests that infrared thermography is capable of identifying local temperature increases associated with diabetic foot infection and other diabetic foot complications.

A temperature difference of approximately 2.2 °C between corresponding contralateral foot regions was consistently reported as a clinically relevant threshold for detecting diabetic foot pathology [11,18].

However, the limited available evidence does not support a correlation between thermographic findings and infection severity, disease progression, or treatment response.

## 4. Discussion

Diabetic foot infection (DFI) remains one of the most challenging complications of diabetes mellitus and is a major cause of hospitalisation, lower-extremity amputation, and increased healthcare expenditure worldwide [6,7]. Early identification of infection is essential to optimise treatment outcomes and prevent progression to severe tissue damage. Consequently, there is growing interest in complementary diagnostic tools capable of identifying pathological changes before overt clinical manifestations become evident. The findings of the present systematic review suggest that infrared thermography may represent a promising adjunctive tool for the diagnosis of DFI through the detection of local temperature changes associated with inflammatory and infectious processes.

As summarised in Table 3, all included studies investigated the use of thermographic assessment in patients with diabetic foot complications, although substantial heterogeneity existed regarding study design, patient characteristics, thermographic equipment, and evaluated outcomes. Despite these differences, the majority of studies reported an association between increased local temperature and diabetic foot complications, supporting the potential role of infrared thermography as an adjunctive diagnostic modality.

Hazenberg et al. [17] demonstrated that infrared thermography combined with photographic assessment provided a valid and reliable method for identifying signs of diabetic foot infection. These findings are particularly relevant in patients with peripheral neuropathy or foot deformities, where clinical examination may be more challenging and classical signs of infection may be less evident. Early recognition of infection in this patient population is essential because delayed diagnosis may increase the risk of hospitalisation, surgical intervention, and amputation.

One of the most clinically relevant findings identified in this review was the repeated observation of thermal asymmetry between corresponding regions of the contralateral feet. Van Netten et al. [18] established a temperature difference of 2.2 °C as the optimal threshold for detecting diabetes-related foot complications. Similarly, Liu et al. [11] reported that this threshold successfully identified 95% of diabetic foot ulcers in their study population. The consistency of these findings across independent studies suggests that thermal asymmetry may represent a sensitive marker of pathological processes occurring in the diabetic foot.

The use of thermal asymmetry analysis offers several practical advantages. Unlike visual clinical assessment, which may be influenced by examiner experience and interobserver variability, thermography provides objective and quantifiable data regarding local inflammatory activity. Furthermore, advances in infrared imaging technology and automated image analysis may facilitate the development of standardised diagnostic algorithms capable of supporting clinical decision-making. Such approaches may be particularly useful in specialised diabetic foot units, multidisciplinary clinics, and telemedicine programmes, where rapid and reproducible assessment methods are required.

Beyond its potential diagnostic role, thermography may also contribute to the identification of diabetic foot complications before the development of clinically evident ulceration. Van Netten et al. [20] demonstrated that patients presenting local complications showed temperature increases greater than 2 °C, whereas diffuse complications such as infected ulcers, Charcot neuroarthropathy, or critical limb ischaemia were associated with temperature differences exceeding 3 °C. These observations support the hypothesis that thermal alterations may reflect disease severity and suggest that thermography could have a role in risk stratification and early intervention strategies.

Nevertheless, the usefulness of thermography for monitoring infection severity and treatment response remains uncertain. Hutting et al. [21] found no significant association between plantar thermal asymmetry and either infection severity or ulcer progression in patients with infected diabetic foot ulcers. Similarly, Armstrong et al. [19] reported no correlation between baseline skin temperature differences and systemic inflammatory markers, including leukocyte count, C-reactive protein levels, and erythrocyte sedimentation rate. Although these findings do not invalidate the diagnostic value of thermography, they suggest that temperature measurements alone may be insufficient for monitoring treatment response and should be interpreted in conjunction with clinical, microbiological, and laboratory findings.

Several advantages make thermography an attractive diagnostic modality in diabetic foot care. The technique is non-invasive, painless, rapid, and does not expose patients to ionising radiation. In addition, repeated measurements can be performed without risk, making thermography suitable for longitudinal assessment and remote monitoring applications. The increasing availability of portable infrared devices may further facilitate the implementation of thermographic assessment in both specialised diabetic foot units and community healthcare settings.

However, several limitations currently restrict the widespread adoption of thermography in clinical practice. One of the most important challenges is the lack of standardised acquisition and interpretation protocols. Environmental conditions, including room temperature, humidity, and patient acclimatisation time, may influence thermographic measurements and affect reproducibility. Furthermore, physiological variability between individuals, differences in peripheral perfusion, and variations in gait patterns may contribute to measurement variability. These factors highlight the need for rigorous standardisation before thermography can be routinely incorporated into clinical guidelines.

Another important limitation is the relatively low specificity of thermographic findings. Although increased temperature may indicate infection, similar thermal alterations can occur in other inflammatory conditions, including mechanical overload, acute Charcot neuroarthropathy, trauma, and local tissue irritation. Consequently, thermographic findings should not be considered diagnostic in isolation but rather as part of a comprehensive clinical assessment that includes patient history, physical examination, microbiological investigations, and complementary imaging when appropriate.

The design-specific risk-of-bias assessments identified several limitations that should be considered when interpreting the available evidence. Among the diagnostic and detection studies, the main concerns involved convenience or purposive sampling, data-driven selection of thermal thresholds, development and evaluation of algorithms within the same study population, and limitations in the clinical reference standards used to establish diabetic foot infection. Applicability was also restricted because most studies included heterogeneous diabetic foot complications rather than populations composed exclusively of patients with diabetic foot infection.

The monitoring studies also presented important methodological limitations. Although Armstrong et al. [19] included a large multicentre sample and used standardised thermometry procedures, the analysis was secondary, potential confounding factors were incompletely addressed, and temperature measurements were not obtained at completion of the full treatment course. Hutting et al. [21] used clearly defined clinical and thermographic procedures, but its convenience sample of only seven participants substantially limited the precision and generalisability of the findings. Collectively, these limitations reduce confidence in the current estimates and reinforce the need for prospective, adequately powered studies with consecutive recruitment, prespecified thermal thresholds, independent validation cohorts, and appropriate clinical reference standards.

The present review also has several limitations. First, only six studies met the eligibility criteria, reflecting the limited amount of available evidence regarding thermography in DFI. Second, substantial heterogeneity existed among the included studies in terms of study design, participant characteristics, thermographic equipment, acquisition protocols, and outcome measures. Third, all included studies were classified as level 4 evidence with a grade of recommendation C, indicating a generally low level of evidence.

Despite these limitations, this review provides a comprehensive synthesis of the current evidence regarding the role of thermography in diabetic foot infection. The available literature supports the potential diagnostic value of thermal assessment, particularly through the evaluation of temperature asymmetry between contralateral foot regions. Nevertheless, further high-quality prospective studies involving larger patient cohorts are required to establish standardised temperature thresholds, validate diagnostic algorithms, and determine the clinical and economic impact of incorporating thermography into diabetic foot management programmes.

Future research should focus on multicentre prospective studies with standardised acquisition protocols and robust methodological designs. Additionally, the integration of thermographic imaging with artificial intelligence, automated image analysis, and remote monitoring technologies may represent a promising avenue for improving the early detection and management of diabetic foot complications. Such developments could contribute to reducing the burden of DFI and ultimately improve patient outcomes.

### Clinical Implications and Future Perspectives

From a clinical perspective, the available evidence suggests that infrared thermography may be most useful during the early evaluation of patients with suspected diabetic foot infection, when increased local skin temperature or thermal asymmetry may support clinical suspicion before overt inflammatory signs become evident. In this context, thermography should be considered an adjunctive assessment tool rather than a standalone diagnostic method.

Conversely, the current evidence does not support the routine use of infrared thermography for monitoring infection severity, treatment response, or disease progression. The available studies found no consistent relationship between thermographic findings and inflammatory markers, infection severity, or clinical evolution.

Furthermore, all included studies were conducted in outpatient or specialised diabetic foot settings. Consequently, there is currently insufficient evidence to recommend the routine use of thermography in emergency departments, inpatient settings, or patients with severe diabetic foot infections. Future prospective studies should evaluate its clinical utility across different healthcare settings and according to validated diabetic foot infection severity classifications, such as the IWGDF/IDSA system.

## 5. Conclusions

Infrared thermography appears to be a promising adjunctive tool for the early detection of diabetic foot infection by identifying increases in local skin temperature and thermal asymmetry between corresponding regions of the feet. Current evidence suggests that thermal asymmetry, particularly temperature differences of approximately 2.2 °C, may assist in the early recognition of diabetic foot pathology and support clinical assessment.

However, the available evidence is limited and heterogeneous, and there is currently insufficient evidence to support the use of infrared thermography for monitoring infection severity, disease progression, treatment response, or prognosis. Therefore, thermography should be considered an adjunct to, rather than a replacement for, comprehensive clinical assessment. Further well-designed prospective studies using standardised acquisition protocols and independent validation are required before thermography can be recommended for routine monitoring of diabetic foot infection.

## Figures and Tables

**Table 1:** PICO framework used to formulate the research question.

P (Population)	Patients with diabetic foot infection
I (Intervention)	Diagnosis of diabetic foot infection
C (Comparison)	Use of thermography as a diagnostic tool versus no thermographic assessment
O (Outcomes)	Early detection of infection

**Table 2:** Characteristics of included studies.

Author	Year	N	Design	Thermography	Main Findings
Hazenberg [17]	2014	38	Observational	Infrared thermometer	Valid and reliable for DFI assessment
Liu [11]	2015	76	Observational	Infrared thermography	2.2 °C threshold detected 95% of ulcers
Van Netten [18]	2014	54	Observational	Infrared camera	2.2 °C useful for diabetic foot complications
Armstrong [19]	2006	362	Observational	Infrared thermometer	No correlation with inflammatory markers
Van Netten [20]	2013	15	Pilot	Infrared camera	>2 °C local and >3 °C diffuse complications.
Hutting [21]	2020	7	Pilot	Infrared thermography	No relation with severity/progression.

**Table 3:** Methodological appraisal of studies evaluating infrared thermography for monitoring diabetic foot infection.

Study	Study Design and Appraisal Tool	Methodological Strengths	Main Methodological Concerns
Armstrong et al., 2006 [19]	Prospective cohort analysis; JBI Critical Appraisal Checklist for Cohort Studies	Large multicentre sample; standardized dermal thermometry protocol; centralised investigator training; 92% of participants with baseline thermometry had a follow-up measurement; clinical outcomes assessed within the SIDESTEP trial protocol	Secondary analysis not primarily designed to evaluate thermometry; incomplete identification and control of confounding factors; thermometry not repeated at completion of the full treatment course; exploratory 10 °F threshold without independent validation
Hutting et al., 2020 [21]	Prospective case series; JBI Critical Appraisal Checklist for Case Series	Clearly defined eligibility criteria; standardised thermographic acquisition; DFI severity assessed using IWGDF criteria; detailed reporting of clinical characteristics, inflammatory markers, and outcomes	Convenience sampling; recruitment not demonstrated to be consecutive or complete; sample limited to seven participants; restricted inclusion criteria and limited generalisability

JBI, Joanna Briggs Institute; DFI, diabetic foot infection; IWGDF, International Working Group on the Diabetic Foot. JBI checklist items were interpreted individually, and no overall numerical quality score or arbitrary cut-off was used. Complete item-level assessments are provided in Supplementary Tables S2 and S3.

**Table 4:** Level of evidence, grade of recommendation, SCIE quartile and impact factor.

Reference	Level	Grade	SCIE	Impact Factor
Hazenberg et al. (2014) [17]	4	C	Q1	3.609
Liu et al. (2015) [11]	4	C	Q2	0.688
Van Netten et al. (2014) [18]	4	C	Q1	3.609
Armstrong et al. (2006) [19]	4	C	Q1	0.877
Van Netten et al. (2013) [20]	4	C	Q1	1.397
Hutting et al. (2020) [21]	4	C	-	-

**Table 5:** Characteristics of the studies included in the systematic review. CRP, C-reactive protein; ESR, erythrocyte sedimentation rate; DFU, diabetic foot ulcer.

Study	Year	Sample (n)	Design	DF Classification	Thermographic Modality	Evaluated Parameter s	Objective	Main Findings
Hazenberg et al. [17]	2014	38	Observational	PEDIS	Infrared thermometer	Infection grade, ulcer depth, callus, hyperkeratosis, blisters and fissures	To determine the validity and reliability of infrared thermography for identifying diabetic foot infection	Combining thermographic findings with photographic assessment Improved identification of diabetic foot infection.
Liu et al. [11]	2015	76	Observational	Not reported	Infrared thermography	Temperature differences between contralateral feet	To investigate whether contralateral thermal asymmetry could identify diabetic foot complications	A temperature difference ≥2.2 °C identified diabetic foot lesions with 95% sensitivity.
Van Netten et al. [18]	2014	54	Observational	PEDIS	Infrared camera	Mean temperature difference between corresponding regions	To evaluate thermal asymmetry as an indicator of diabetic foot complications requiring urgent treatment	A cut-off of 1.35 °C showed optimal diagnostic performance, although overall diagnostic Accuracy remained limited.
Armstrong et al. [19]	2006	362	Observational	Texas	Infrared thermometer	Leukocyte count, CRP, ESR and initial thermal asymmetry	To investigate the relationship between thermographic findings and inflammatory markers of diabetic foot infection	Initial thermal asymmetry differed according to ulcer severity; however, no significant correlation was found between thermographic findings and inflammatory markers associated with diabetic foot infection.
Van Netten et al. [20]	2013	15	Pilot study	Texas	Infrared camera	Thermal patterns in healthy feet and diabetic foot complications	To explore the feasibility of automated infrared image analysis for detecting diabetic foot abnormalities	Patients with diabetic foot complications showed greater thermal asymmetry than healthy controls, particularly in Charcot neuroarthropathy.
Hutting et al. [21]	2020	7	Pilot case series	IWGDF/I DSA	Infrared thermography	Plantar thermal asymmetry during treatment	To evaluate whether plantar thermal asymmetry reflects infection severity during treatment	No relationship was observed between plantar thermal asymmetry and diabetic foot infection severity or progression, indicating that thermography is not suitable for monitoring treatment response, although it may assist in early detection.

## Data Availability

The data that support the findings of this study are available from the corresponding author upon reasonable request.

## Supplementary Materials

The following supporting information can be downloaded at https://www.mdpi.com/article/10.3390/idr18050098/s1: Table S1. PRISMA 2020 Checklist [16]. Table S2: JBI critical appraisal of Armstrong et al. [19]; Table S3: JBI critical appraisal of Hutting et al. [21]; Table S4: Domain-level QUADAS-2 assessment of diagnostic and detection studies.
