# Supplementary material for: The Role of Infrared Thermography in the Diagnosis and Monitoring of Diabetic Foot Infection: A Systematic Review
Source: Infect Dis Rep. 2026 Sep 7;18(5):98. doi: 10.3390/idr18050098 (PMC13600055; doi:10.3390/idr18050098)
Supplement: Supplementary file 1 [file idr-18-00098-s001.zip › idr-4380562-supplementary.pdf]

Supplementary Table S1. PRISMA 2020 Checklist [16]

Section and Topic	Item #	Checklist item	Location where item is reported
**TITLE**			
Title	1	Identify the report as a systematic review.	Title page
**ABSTRACT**			
Abstract	2	See the PRISMA 2020 for Abstracts checklist.	Abstract
**INTRODUCTION**			
Rationale	3	Describe the rationale for the review in the context of existing knowledge.	Introduction, paragraphs 1–6
Objectives	4	Provide an explicit statement of the objective(s) or question(s) the review addresses.	End of Introduction; Section 2.1
**METHODS**			
Eligibility criteria	5	Specify the inclusion and exclusion criteria for the review and how studies were grouped for the syntheses.	Section 2.3
Information sources	6	Specify all databases, registers, websites, organisations, reference lists and other sources searched or consulted to identify studies. Specify the date when each source was last searched or consulted.	Section 2.2
Search strategy	7	Present the full search strategies for all databases, registers and websites, including any filters and limits used.	Section 2.2
Selection process	8	Specify the methods used to decide whether a study met the inclusion criteria of the review, including how many reviewers screened each record and each report retrieved, whether they worked independently, and if applicable, details of automation tools used in the process.	Section 2.4
Data collection process	9	Specify the methods used to collect data from reports, including how many reviewers collected data from each report, whether they worked independently, any processes for obtaining or confirming data from study investigators, and if applicable, details of automation tools used in the process.	Section 2.5
Data items	10a	List and define all outcomes for which data were sought. Specify whether all results that were compatible with each outcome domain in each study were sought (e.g. for all measures, time points, analyses), and if not, the methods used to decide which results to collect.	Section 2.8
	10b	List and define all other variables for which data were sought (e.g. participant and intervention characteristics, funding sources). Describe any assumptions made about any missing or unclear information.	Section 2.5
Study risk of bias assessment	11	Specify the methods used to assess risk of bias in the included studies, including details of the tool(s) used, how many reviewers assessed each study and whether they worked independently, and if applicable, details of automation tools used in the process.	Section 2.6
Effect measures	12	Specify for each outcome the effect measure(s) (e.g. risk ratio, mean difference) used in the synthesis or presentation of results.	Not applicable (not meta-analysis performed)
Synthesis methods	13a	Describe the processes used to decide which studies were eligible for each synthesis (e.g. tabulating the study intervention characteristics and comparing against the planned groups for each synthesis (item #5)).	Section 2.3, 2.8
	13b	Describe any methods required to prepare the data for presentation or synthesis, such as handling of missing summary statistics, or data conversions.	Not reported
	13c	Describe any methods used to tabulate or visually display results of individual studies and syntheses.	Tables 2-5
	13d	Describe any methods used to synthesize results and provide a rationale for the choice(s). If meta-analysis was performed, describe the model(s), method(s) to identify the presence and extent of statistical heterogeneity, and software package(s) used.	Section 2.8
	13e	Describe any methods used to explore possible causes of heterogeneity among study results (e.g. subgroup analysis, meta-regression).	Not performed
	13f	Describe any sensitivity analyses conducted to assess robustness of the synthesized results.	Nor performed

Section and Topic	Item #	Checklist item	Location where item is reported
Reporting bias assessment	14	Describe any methods used to assess risk of bias due to missing results in a synthesis (arising from reporting biases).	Not reported
Certainty assessment	15	Describe any methods used to assess certainty (or confidence) in the body of evidence for an outcome.	Section 2.7, 3.2
**RESULTS**			
Study selection	16a	Describe the results of the search and selection process, from the number of records identified in the search to the number of studies included in the review, ideally using a flow diagram.	Section 3.1; Figure 1
	16b	Cite studies that might appear to meet the inclusion criteria, but which were excluded, and explain why they were excluded.	Section 3.1
Study characteristics	17	Cite each included study and present its characteristics.	Section 3.2; Table 3
Risk of bias in studies	18	Present assessments of risk of bias for each included study.	Section 3.6; Tables 4–5
Results of individual studies	19	For all outcomes, present, for each study: (a) summary statistics for each group (where appropriate) and (b) an effect estimate and its precision (e.g. confidence/credible interval), ideally using structured tables or plots.	Sections 3.3–3.5; Table 3
Results of syntheses	20a	For each synthesis, briefly summarise the characteristics and risk of bias among contributing studies.	Sections 3.3–3.7
	20b	Present results of all statistical syntheses conducted. If meta-analysis was done, present for each the summary estimate and its precision (e.g. confidence/credible interval) and measures of statistical heterogeneity. If comparing groups, describe the direction of the effect.	Not applicable (no meta-analysis performed)
	20c	Present results of all investigations of possible causes of heterogeneity among study results.	Not performed
	20d	Present results of all sensitivity analyses conducted to assess the robustness of the synthesized results.	Not performed
Reporting biases	21	Present assessments of risk of bias due to missing results (arising from reporting biases) for each synthesis assessed.	Not reported
Certainty of evidence	22	Present assessments of certainty (or confidence) in the body of evidence for each outcome assessed.	Section 2.7, 3.2
**DISCUSSION**			
Discussion	23a	Provide a general interpretation of the results in the context of other evidence.	Section 4
	23b	Discuss any limitations of the evidence included in the review.	Section 4
	23c	Discuss any limitations of the review processes used.	Section 4
	23d	Discuss implications of the results for practice, policy, and future research.	Section 4; Section 5
**OTHER INFORMATION**			
Registration and protocol	24a	Provide registration information for the review, including register name and registration number, or state that the review was not registered.	Not reported

Section and Topic	Item #	Checklist item	Location where item is reported
	24b	Indicate where the review protocol can be accessed, or state that a protocol was not prepared.	Not reported
	24c	Describe and explain any amendments to information provided at registration or in the protocol.	Not applicable
Support	25	Describe sources of financial or non-financial support for the review, and the role of the funders or sponsors in the review.	Acknowledgments
Competing interests	26	Declare any competing interests of review authors.	Conflicts of Interest
Availability of data, code and other materials	27	Report which of the following are publicly available and where they can be found: template data collection forms; data extracted from included studies; data used for all analyses; analytic code; any other materials used in the review.	Data Availability Statement

From: Page MJ, McKenzie JE, Bossuyt PM, Boutron I, Hoffmann TC, Mulrow CD, et al. The PRISMA 2020 statement: an updated guideline for reporting systematic reviews. BMJ 2021;372:n71. doi: 10.1136/bmj.n71. This work is licensed under CC BY 4.0. To view a copy of this license, visit https://creativecommons.org/licenses/by/4.0/

Supplementary Table S2. JBI critical appraisal of Armstrong et al. [19]

JBI cohort checklist item	Judgment	Rationale
Were the groups similar and recruited from the same population?	N/A	Temperature was analysed primarily as a continuous exposure; distinct exposed and unexposed cohorts were not defined prospectively.
Were exposures measured similarly to assign participants to exposed and unexposed groups?	Yes	The same thermometry procedure was used for all participants.
Was the exposure measured in a valid and reliable way?	Yes	Investigators received centralised training and used a standardised infrared thermometer and protocol.
Were confounding factors identified?	No	Relevant factors such as peripheral arterial disease, previous amputation, wound location, and wound characteristics were not comprehensively addressed.
Were strategies to deal with confounding factors stated?	No	Adjustment was limited and no comprehensive multivariable analysis was reported for the relationship between temperature and clinical response.
Were participants free of the outcome at the start of the study?	Yes	Clinical response to treatment had not occurred at baseline.
Were outcomes measured in a valid and reliable way?	Yes	Clinical outcomes were assessed using standardised procedures within the SIDESTEP trial.
Was follow-up time reported and sufficient for outcomes to occur?	No	Thermometry was repeated at discontinuation of intravenous therapy but not at completion of the full treatment course.
Was follow-up complete, and were reasons for loss to follow-up described and explored?	Yes	Follow-up thermometry was available for 332 of 362 participants with baseline measurements.
Were strategies used to address incomplete follow-up?	No	No sensitivity analysis, imputation, or comparison of participants with and without follow-up thermometry was reported.
Was appropriate statistical analysis used?	Unclear	Paired analyses, correlations, and ANCOVA were used, but the 10 °F threshold analysis was exploratory and insufficiently adjusted.

Supplementary Table S3. JBI critical appraisal of Hutting et al. [21]

JBI case-series checklist item	Judgment	Rationale
Were there clear criteria for inclusion in the case series?	Yes	Eligibility and exclusion criteria were clearly specified.
Was the condition measured in a standard and reliable way for all participants?	Yes	DFI severity was assessed using standard IWGDF infection grades.
Were valid methods used for identification of the condition for all participants?	Yes	Clinical criteria were consistently applied and supported by inflammatory markers and osteomyelitis assessment when required.
Did the case series have consecutive inclusion of participants?	No	The authors explicitly described the sample as a convenience sample.
Did the case series have complete inclusion of participants?	No	It was not demonstrated that all eligible patients during the recruitment period were included.
Was there clear reporting of participant demographics?	Yes	Relevant demographic and baseline characteristics were reported.
Was there clear reporting of clinical information?	Yes	DFI severity, PAD, neuropathy, osteomyelitis, treatment, and inflammatory markers were described.
Were outcomes or follow-up results clearly reported?	Yes	Thermal asymmetry, infection grade, inflammatory markers, and clinical outcomes were reported.

Supplementary Table S4. Domain-level QUADAS-2 assessment of diagnostic and detection studies

Study	Patient selection: risk of bias	Index test: risk of bias	Reference standard: risk of bias	Flow and timing: risk of bias	Patient selection: applicability	Index test: applicability	Reference standard: applicability
Hazenberg et al., 2014 [16]	Low	Low	High	Low	High concern	Low concern	Low concern
van Netten et al., 2014 [17]	High	High	Low	Low	High concern	Low concern	Low concern
Liu et al., 2015 [11]	High	High	High	Low	High concern	High concern	High concern
van Netten et al., 2013 [19]	High	High	Low	Low	High concern	Low concern	Low concern

QUADAS-2, Quality Assessment of Diagnostic Accuracy Studies-2. Judgements were based on the signalling questions and domain-level guidance of the QUADAS-2 tool.
